# Methotrexate inhibits SARS‐CoV‐2 virus replication “in vitro”

**DOI:** 10.1002/jmv.26512

**Published:** 2020-09-28

**Authors:** Arnaldo Caruso, Francesca Caccuri, Antonella Bugatti, Alberto Zani, Marco Vanoni, Paolo Bonfanti, Marina E. Cazzaniga, Carlo F. Perno, Cristina Messa, Lilia Alberghina

**Affiliations:** ^1^ Department of Molecular and Translational Medicine, Section of Microbiology and Virology University of Brescia Medical School Brescia Italy; ^2^ Department of Biotechnology and Biosciences, ISBE.IT‐SYSBIO/Centre of Systems Biology University of Milano Bicocca Milano Italy; ^3^ School of Medicine and Surgery University of Milano Bicocca Milano Italy; ^4^ Clinic of Infectious Diseases—ASST Monza Monza Italy; ^5^ ASST Monza, Phase 1 Research Unit Monza Italy; ^6^ Irccs Children Hospital Bambino Gesù Rome Italy; ^7^ Tecnomed Foundation Milan Italy

**Keywords:** COVID‐19, drug repurposing, methotrexate, purine biosynthesis inhibition

## Abstract

In early 2020 the new respiratory syndrome COVID‐19 (caused by the zoonotic SARS‐CoV‐2 virus) spread like a pandemic, starting from Wuhan, China, causing severe economic depression. Despite some advances in drug treatments of medical complications in the later stages of the disease, the pandemic's death toll is tragic, as no vaccine or specific antiviral treatment is currently available. By using a systems approach, we identify the host‐encoded pathway, which provides ribonucleotides to viral RNA synthesis, as a possible target. We show that methotrexate, an FDA‐approved inhibitor of purine biosynthesis, potently inhibits viral RNA replication, viral protein synthesis, and virus release. The effective antiviral methotrexate concentrations are similar to those used for established human therapies using the same drug. Methotrexate should be most effective in patients at the earliest appearance of symptoms to effectively prevent viral replication, diffusion of the infection, and possibly fatal complications.

## Highlights

- The COVID‐19 pandemic caused by the SARS‐CoV‐2 virus is a global public health threat causing over 800000 deaths, millions of infected people, and severe economic depression.
- No vaccine or specific antiviral treatments are currently available.
- By inhibiting the host‐encoded pathway, which provides ribonucleotides to viral RNA synthesis, the FDA‐approved drug methotrexate efficiently blocks SARS‐CoV‐2 replication.

## 1 INTRODUCTION

Due to the massive diffusion of the COVID‐19 pandemic, the present worldwide health emergency, with over 845,000 deaths and millions of infected people, is severely disrupting social and economic activities. If no efficient treatment or specific vaccine is soon available worldwide, the occurrence of a severe global recession, which would bring significant social and economic turmoil, is feared in the future.

COVID‐19 is an infectious respiratory disease caused by SARS‐CoV‐2, a new zoonotic virus that undergoes mutational variability.^1^ Inhalation of virus‐containing respiratory droplets and airborne transmission represents the most relevant infective routes.^2^ The vast majority of infected people are asymptomatic or present mild health problems. Asymptomatic and symptomatic patients may present a comparable viral load, and both may be infective.^3^ The median incubation period (from infection to symptom onset) is 5.1 days. Over 97% of symptomatic patients develop signs of disease within 11.5 days.^4^

If the invading SARS‐CoV‐2 virus elicits a strong protective immune response, the patient shows non‐severe symptoms. If the immune system fails to eliminate the virus, the patient develops viral pneumonia symptoms, with dyspnea, which may lead to severe and potentially lethal complications.^5^, ^6^, ^7^ This more dangerous stage is characterized by a dysfunctional immune response that leads to a cytokine storm, which predisposes to hemostatic abnormalities.^5^, ^6^, ^7^ High levels of interleukin‐6 (IL‐6), together with significant levels of SARS‐CoV‐2 RNA, are an index of a high lethality risk. In the most severe cases, acute respiratory distress syndrome (ARDS), often complicated by pulmonary thromboembolism, occurs finally, leading to death.^8^, ^9^ Extension of the viral infection to extrapulmonary districts^10^ may have a role in the diffused post‐COVID syndrome.^11^

Patients with mild symptoms receive mostly symptomatic treatments.^6^ Patients in the severe stage receive anti‐inflammatory drugs, including low doses of steroids or drugs acting on the IL‐6 axis.^12^ As the COVID‐19 pandemic would be substantially constrained by a drug blocking the reproduction of viral particles, several studies are ongoing^12^ to reposition available drugs looking for their ability to interfere with essential viral functions.

The novelty of the present report is provided by considering, in a system biology approach, not only the events of viral entrance and reproduction into lung type II pneumocytes but also their necessary collaboration with the human host cellular pathways, among which the one providing nucleotides required for the synthesis of viral RNA. The synthesis of purines (a component of nucleotides) is inhibited by methotrexate (4‐amino‐10‐methyl folic acid, MTX),^13^ an FDA approved drug, used to treat several cancer types and, at lower doses, autoimmune diseases. Herein, we present the first round of experiments "in vitro," which clearly show that MTX blocks, with high efficiency, SARS‐CoV‐2 replication in the post‐entry phase.

## 2 MATERIALS AND METHODS

### 2.1 Cells

African green monkey kidney Vero E6 cell line was obtained from American Type Culture Collection (ATCC) and maintained in Dulbecco's modified Eagle's medium (DMEM; Gibco, Thermo Fisher Scientific) supplemented with 10% fetal bovine serum (FBS; Gibco, Thermo‐Fisher Scientific) at 37°C in a humidified atmosphere of 5% CO_2_.

### 2.2 Virus

We successfully isolated SARS‐CoV‐2 in Vero E6 cells from the nasopharyngeal swab sample of a COVID‐19 patient. The identity of the strain was verified in Vero E6 cells by real‐time polymerase chain reaction (PCR) and by metagenomic sequencing, from which the reads mapped to nCoV‐2019 (genomic data are available at EBI under study accession n. PRJEB38101). We propagated the clinical isolate in Vero E6 cells and determined the viral titer by a standard plaque assay. We performed infection experiments in a biosafety level‐3 (BLS‐3) laboratory at a multiplicity of infection (MOI) of 0.05 and 1.0.

### 2.3 Efficacy study of MTX

Vero E6 cells were seeded at a density of 5 × 10^4^ cells/well in a 24‐well plate and treated with different doses of MTX (Sigma‐Aldrich). The efficacy of MTX was determined by CellTiter‐Glo (Promega) that measures the ATP levels and direct counting of the viable cell number after trypan‐blue staining.

### 2.4 Evaluation of antiviral efficacy of methotrexate

Vero E6 cells were infected for one h with the SARS‐CoV‐2 isolate at an MOI of 0.05. Infection was carried out in DMEM medium without FBS. Then, after virus removal and washing with warm phosphate‐buffered saline (we cultured cells in a medium containing 2% FBS in the presence or the absence of MTX at the concentration of 25, 2.5, or 0.25 μM. At 48 h postinfection, both cells and supernatants were collected for further viral genome quantification analysis.

### 2.5 Viral RNA extraction and quantitative real‐time reverse‐transcription PCR (qRT‐PCR)

RNA was extracted from clarified cell culture supernatants (16,000*g* × 10 min) and infected cells using a QIAamp Viral RNA Mini Kit and RNeasy Plus mini kit (Qiagen), respectively, according to the manufacturer's instructions.

RNA was eluted in 30 μl of RNase‐free water and stored at –80°C till use. The qRT‐PCR was carried‐out following previously described procedures with minor modifications.^14^ Briefly, reverse transcription and amplification of the S gene were performed using the one‐step QuantiFast Sybr Green RT‐PCR mix (Qiagen) as follows: 50°C for 10 min, 95°C for 5 min; 95°C for 10 s, 60°C for 30 s (40 cycles) (primers: RBD‐qF1: 5’‐CAATGGTTTAACAGGCACAGG‐3’ and RBD‐qR1: 5’‐CTCAAGTGTCTGTGGATCACG‐3). A standard curve was generated by determination of copy numbers derived from serial dilutions (10^3^–10^9^ copies) of a pGEM T‐easy vector (Promega) containing the receptor‐binding domain of the S gene (primers: RBD‐F: 5’‐GCTGGATCCCCTAATATTACAAACTTGTGCC‐3’; RBD‐R: 5’‐TGCCTCGAGCTCAAGTGTCTGTGGATCAC‐3’). Each quantification was run in triplicate.

### 2.6 Western blot analysis

Protein samples (30 µg) obtained from lysis in RIPA buffer (Cell Signaling Technology, Danvers) of Vero E6 infected cells were separated on 10% sodium dodecyl sulphate‐polyacrylamide gel electrophoresis and then transferred onto polyvinylidene difluoride membranes (Millipore, Sigma). After being blocked with 3% bovine serum albumin in tris buffered saline buffer containing 0.05% Tween 20, the blot was probed with a human serum (1:1000 dilution) containing IgG to the SARS‐CoV‐2 nucleoprotein (NP) and with mouse anti‐human GAPDH monoclonal antibody (G‐9; Santa Cruz Biotechnology). The antigen‐antibody complexes were detected using peroxidase‐conjugated goat anti‐human or goat antimouse IgG (Sigma) and revealed using the enhanced chemiluminescence (ECL) system (Santa Cruz Biotechnology).

### 2.7 Statistical analysis

Data were analyzed for statistical significance using the one‐way analysis of variance, and the Bonferroni post test was used to compare data. Differences were considered significant when *p* < .05. Statistical tests were performed using GraphPad Prism 8.

## 3 RESULTS

### 3.1 Effects of methotrexate on Vero E6 cells growth and metabolism

**Figure 1:**
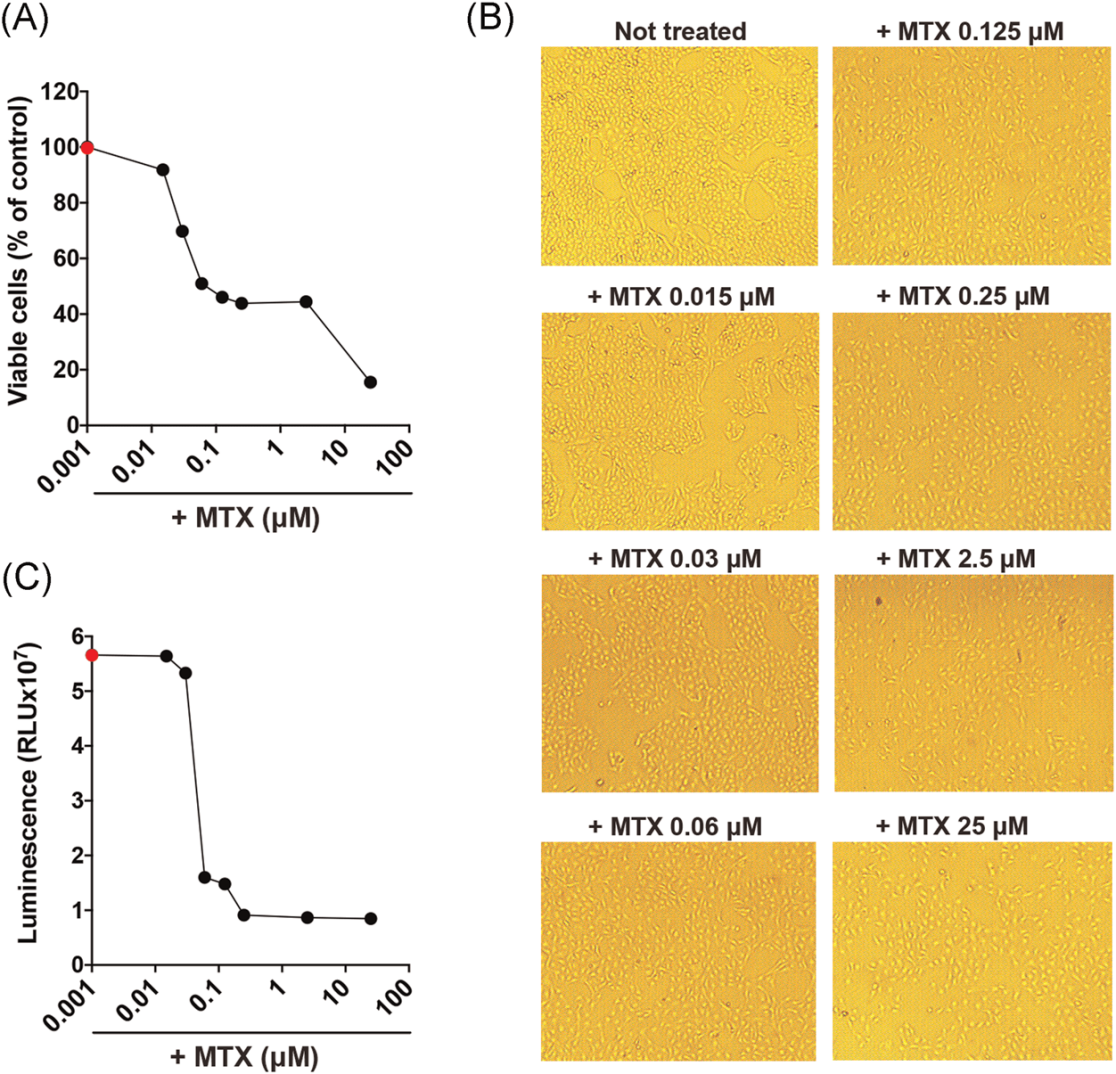
Determination of antimetabolic and proliferative efficacy of MTX. (A) Inhibition of cell growth after 48 h of culture in the presence of MTX at different concentrations (25, 2.5, 0.25, 0.125, 0.06, 0.03, and 0.015 μM). (B) 10× bright‐field images of Vero E6 cells after incubation for 48 h at 37°C with the indicated MTX concentrations with an initial cell density of 5 × 10^4^ cells/well. (C) measurement of ATP by CellTiter‐Glo as a luminescent readout of cell viability. Red dots in (A) and (C) refer to cells not treated with MTX

At first, we assayed the effect of various concentrations of MTX on cell proliferation of Vero E6 cells, an established model system for SARS‐CoV‐2 isolation and replication. After 48 h of culture, 0.015 µM MTX has a negligible effect on the extent of growth, assayed as the viable cell number detected by the Trypan Blue dye exclusion test. At this very low concentration, MTX inhibition may be competitively displaced by the intracellular substrate of DHFR. MTX concentrations ranging from 0.06 to 2.5 µM have a potent inhibitory effect (around 45%) that becomes substantially stronger (85%) at MTX 25 µM (Figure 1A). MTX‐treated cells display a normal surface‐adherent phenotype (Figure 1B).

MTX severely inhibits the accumulation of cellular ATP (assayed with CellTiter‐Glo). For an extensive range of MTX concentrations, intracellular ATP levels' inhibition remains reasonably constant, ranging from 73% inhibition at MTX 0.06 µM to 82% for 25 µM (Figure 1C).

Taken together, these data indicate that at concentrations between 0.06 and 2.5 µM, the proliferative ability of MTX‐treated Vero E6 cells correlates with down‐modulation of their metabolic activity, with negligible effects on cell morphology and minor cytotoxicity.

### 3.2 MTX potently inhibits SARS‐CoV‐2 replication

**Figure 2:**
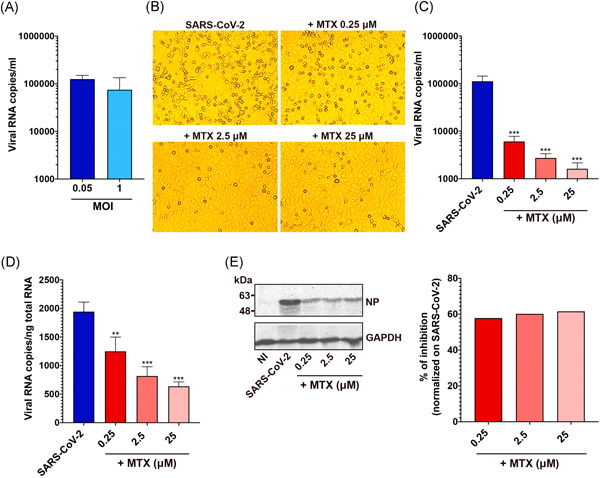
Antiviral activity of MTX. (A) Vero E6 cells were infected with SARS‐CoV‐2 at a multiplicity of infection (MOI) of 0.05 or 1.0 for 1 h at 37°C and then washed and cultured for 48 h. Viral yield was quantitated in the cell supernatant by quantitative real‐time reverse‐transcription PCR (qRT‐PCR). At least three independent replicates were performed. Data are representative of two independent experiments with similar results. In (B)–(E), Vero E6 cells were infected with SARS‐CoV‐2 at an MOI of 0.05 in the absence or in the presence of different doses of MTX. (B) Cells were imaged with an optical microscope to detect typical SARS‐CoV‐2‐induced cytolytic effects. (C) Viral yield was quantitated in the cell supernatant by qRT‐PCR. At least three independent replicates were performed. Data are representative of two independent experiments with similar results. (***p < .001). (D) Quantitation of SARS‐CoV‐2 genomes at the intracellular level by qRT‐PCR. At least three independent replicates were performed. Data are representative of two independent experiments with similar results. (**p < .01; ***p < .001). (E) Nucleoprotein (NP) expression in infected cells was analyzed by Western blot (left panel). Densitometric analysis of Western blot results is shown in the right panel. Bars represent mean % inhibition of NP expression in cells treated with MTX at different concentrations, normalized to control SARS‐CoV‐2‐infected cells

We infected Vero E6 cells at either low MOI (MOI = 0.05) or high MOI (MOI = 1.0). Quantification of released RNA after 48 h indicates that viral production is very similar at both MOIs (Figure 2A). Therefore, in the following experiments, we use an MOI of 0.05.

Next, we assessed whether MTX could affect the replication of the SARS‐CoV‐2 virus. Vero E6 cells were infected with a primary SARS‐CoV‐2 strain isolated in Brescia, Italy, and one h later they were cultured in the absence or the presence of MTX at the concentrations of 25, 2.5, or 0.25 µM. MTX efficiently inhibits viral replication (Figure 2B‐E). All MTX doses abolish the SARS‐CoV‐2 cytolytic effects on Vero E6 cells, with only limited cytopathic effects detectable at the lowest micromolar dose of MTX used (Figure 2B). Quantification of viral RNA copy number in the cell culture supernatants confirms the potent inhibitory effect of MTX on viral particles' production, with a reduction of nearly 70‐fold at 25 µM and about 20‐fold at 0.25 µM, as compared with untreated infected cells (Figure 2C). qRT‐PCR quantification of intracellular SARS‐CoV‐2 RNA in SARS‐CoV‐2‐infected cells shows dose‐dependent inhibition of intracellular SARS‐CoV‐2 genome expression, ranging from 40% to 70% at the tested MTX concentrations (from 0.25 to 25 µM) compared with untreated cells (Figure 2D). Western blot with patient antisera^15^ recognizing the virus NP confirms MTX's efficacy on virus replication. MTX induces a significant (approximate 60%) inhibition of NP accumulation (Figure 2E). As there are many copies of NP for each RNA molecule in the virion, the detected constant immunoreactive band, observed at all MTX concentrations, may mainly reflect the viral protein present at the beginning of the treatment, masking the robust dose‐dependent inhibition observed in viral RNA assays experiments.

## 4 DISCUSSION

Our results indicate that MTX efficiently inhibits viral replication at the post‐entry stages of the SARS‐CoV‐2 infection. Its dose‐dependent antiviral activity is extremely potent. Consistent with data reported for Zika Virus infection^16^ and the established mechanism of action of MTX,^13^ these pieces of data indicate that the cellular purine biosynthetic pathway is a reliable target to inhibit SARS‐CoV‐2 replication. Accordingly, MTX should be most effective in patients at the earliest appearance of symptoms to prevent the synthesis of new viral RNA and, therefore, the formation of virus particles able to extend the infection to contiguous lung alveolar epithelium and extrapulmonary sites^10^ and to spread the infection to other people through virus‐containing droplet emission. As viral RNA has been detected in non‐survivors until the point of death, a correlation between virus persistence and poor disease outcome has been proposed.^17^

In the first 24 h following different routes of administration of 15 mg MTX to rheumatoid arthritis patients, the drug's plasma concentration remains between 0.1 and 1 μM,^18^ that is, within the effective range reported in our study (0.25–2.5 μM). As the metronomic, multiple administration of low MTX doses^19^ may improve drug absorption, the effective doses reported here are compatible with the therapeutically effective (and well‐tolerated) MTX dosage employed in clinical use. These experiments could provide the basis for designing antiviral treatments that are compatible with patient assistance at home, monitored by family doctors under COVID hospitals' supervision.

In rheumatoid arthritis and other inflammatory syndromes, the molecular target of MTX is not DHFR.^13^ MTX decreases the levels of interleukin 6 (IL‐6) and soluble IL‐2 receptor, the reduction in cytokine levels being paralleled by an improvement in clinical indices.^20^ Therefore, in COVID‐19 patients in a more advanced stage, the treatment with MTX is expected to decrease virion production and down‐regulate the IL‐6 pathway, a strategy currently in use in different clinical trials.^12^

## 5 CONCLUSIONS

We show that MTX exerts a dose‐dependent, potent inhibition of SARS‐CoV‐2 replication in model cell lines. As it targets an enzyme essential for viral replication, MTX could be used as a first‐line intervention even against heavily mutated SARS‐CoV‐2 variants or emerging epidemics caused by novel RNA viruses. The potential ability of MTX to down‐regulate the cytokine storm typical of later stages of the disease could contribute to making oral MTX a new and effective antiviral treatment for the COVID‐19 pandemic. MTX may also be useful in patients suffering from the post‐COVID syndrome,^11^ in which systemic permanence and diffusion of the virus play a role.

## CONFLICT OF INTERESTS

The authors declare that there is no conflict of interest.

## AUTHOR CONTRIBUTIONS

Lilia Alberghina conceived the idea that led to the systems identification of MTX as a potential anti‐COVID drug; Arnaldo Caruso planned the in vitro experiments. Francesca Caccuri isolated, expanded, and titrated the primary SARS‐CoV‐2 isolate obtained in Brescia, Italy. Francesca Caccuri and Alberto Zani performed experiments on the antiviral activity of MTX by quantitating viral genome expression in cells and in cell culture supernatants. Antonella Bugatti performed experiments on the metabolic activity of MTX‐treated or MTX‐untreated cells and on the SARS‐CoV‐2 NP antigen expression in cells by Western blot; Paolo Bonfanti, Carlo F. Perno, and Marina E. Cazzaniga evaluated clinical perspectives; Arnaldo Caruso and Francesca Caccuri analyzed in vitro data; Arnaldo Caruso, Marco Vanoni, and Lilia Alberghina collected data and wrote the manuscript; Cristina Messa and Carlo F. Perno assembled, coordinated and guided the multidisciplinary team; all the authors discussed, reviewed and approved the manuscript.

## Data Availability

The data that support the findings of this study are available from the corresponding author upon reasonable request.
